# Supplementary figures and images for: Flor Yeasts Rewire the Central Carbon Metabolism During Wine Alcoholic Fermentation
Source: Front Fungal Biol. 2021 Oct 18;2:733513. doi: 10.3389/ffunb.2021.733513 (PMC10512321; doi:10.3389/ffunb.2021.733513)

Correlation coefficient =  $-0.2$  (pval = 0 )

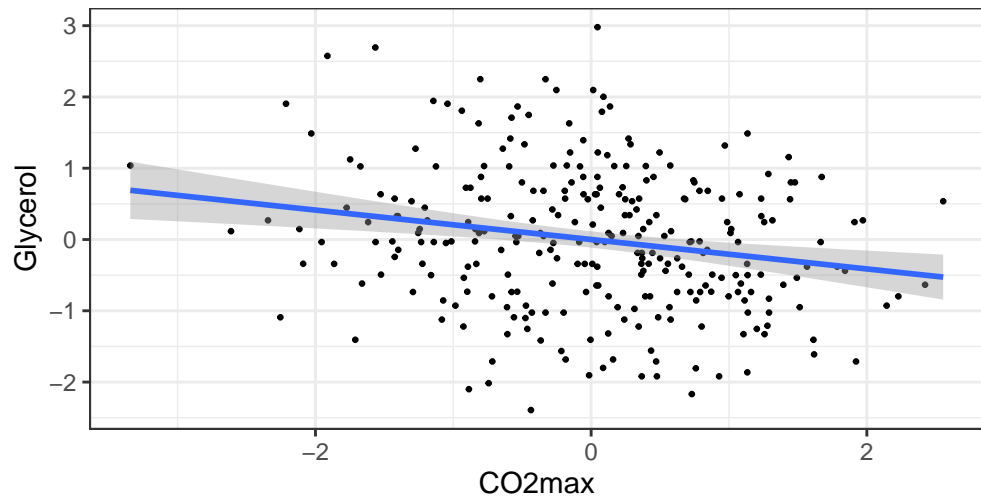

Correlation coefficient =  $0.25$  (pval = 0 )

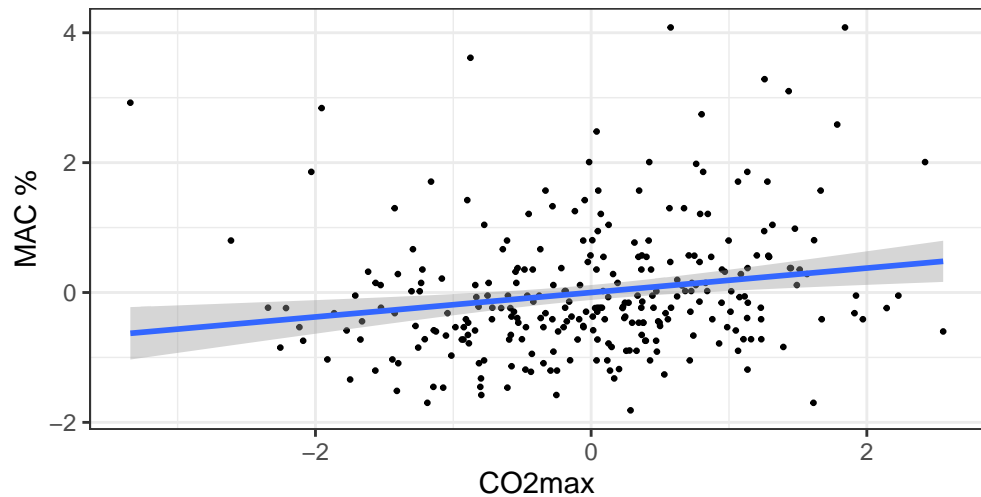

Supplement: Supplementary Figure 1 — Correlation between traits. Data is normalized according to environment. Each dot represents the average value of an individual in one of the three phenotypic condition. Correlation coefficient and P value of Spearman's correlation test is indicated. CO2max is negatively correlated with glycerol and positively correlated with MAC% (Spearman test, p < 0.01). However, rho values observed are quite low (<0.2) because the variation in CO2 production is balanced by glycerol production and malic acid consumption. [file Image_1.PDF]

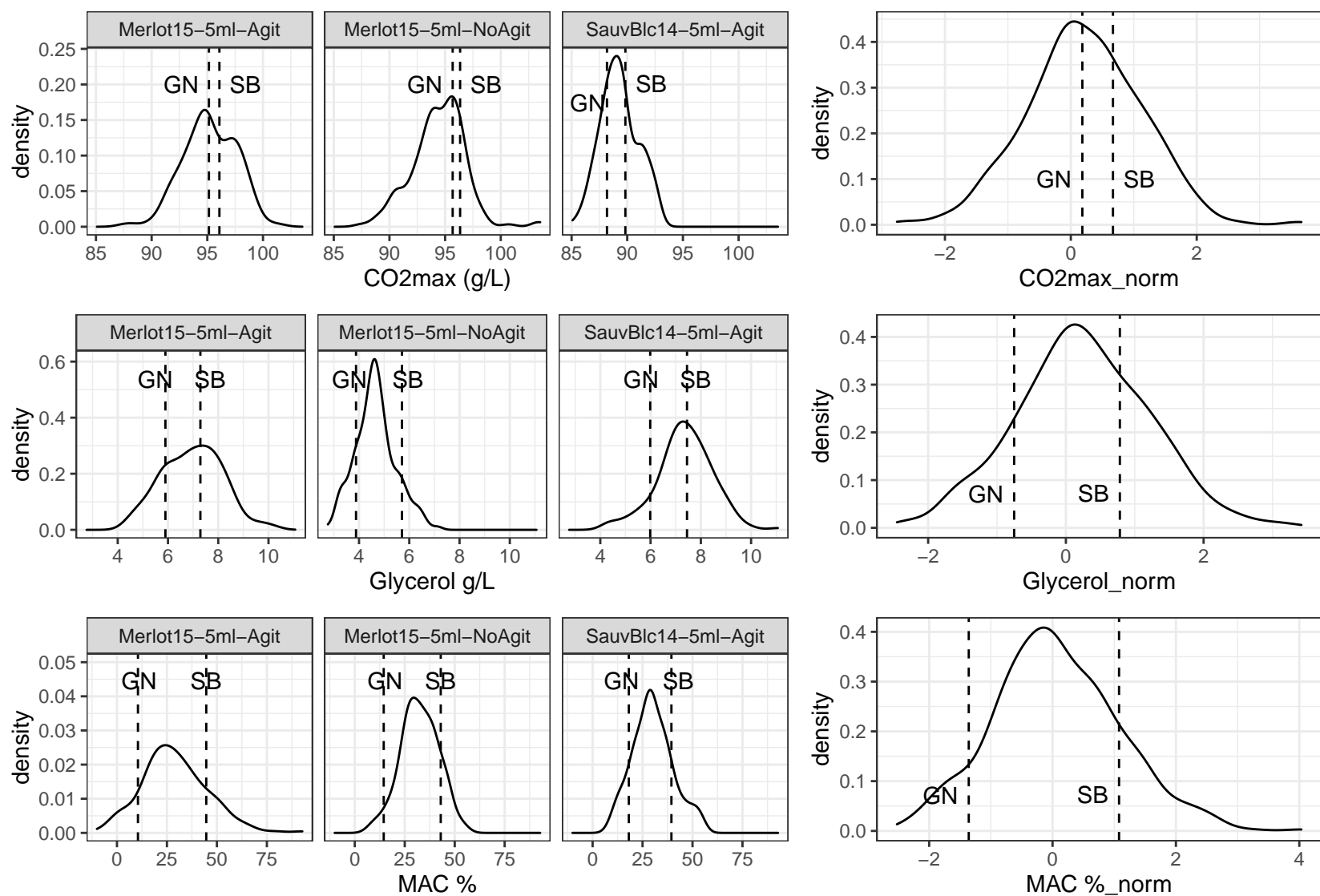

Supplement: Supplementary Figure 2 — Distribution of traits. (Left) Distribution of the progeny according to trait and media is represented. Dashed vertical line represent parental average value. (Right) Data is normalized according to environment. Distribution of the progeny in all media, according to trait and cross. Dashed vertical line represent parental average value. [file Image_2.PDF]

VII\_482

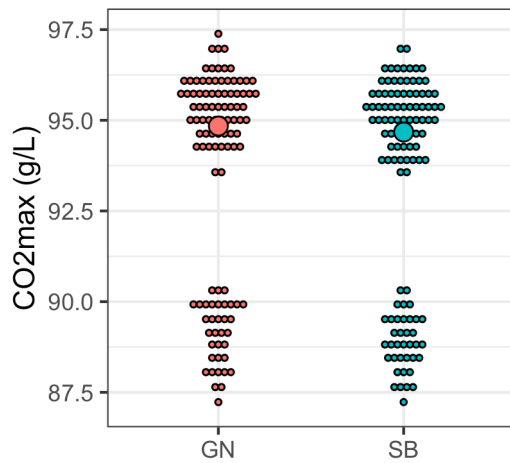

VII\_522

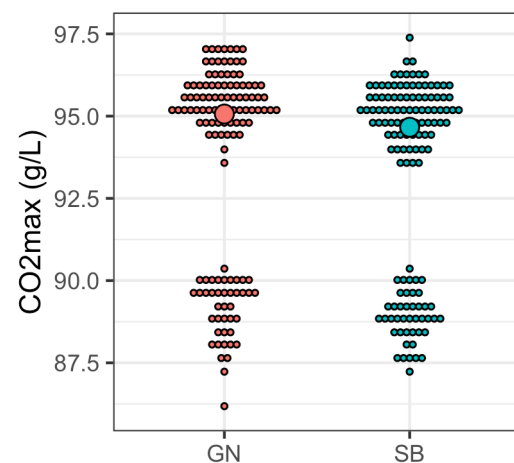

VII\_614

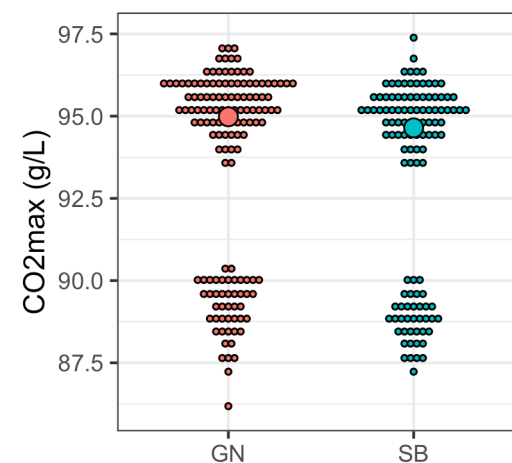

I\_74

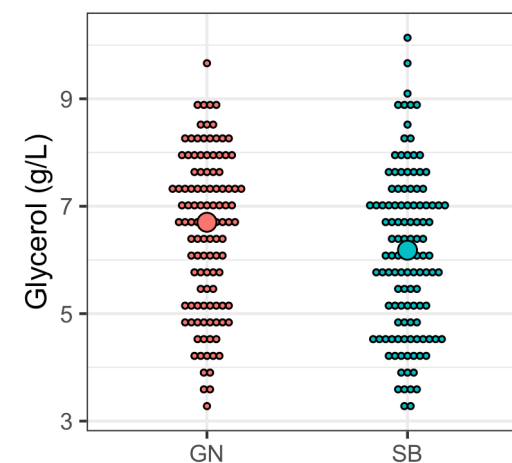

VII\_407

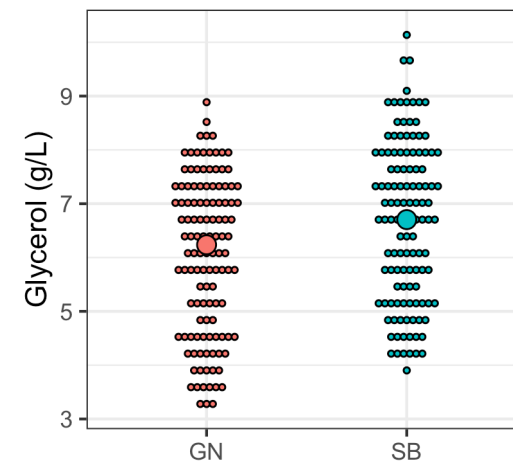

II\_152

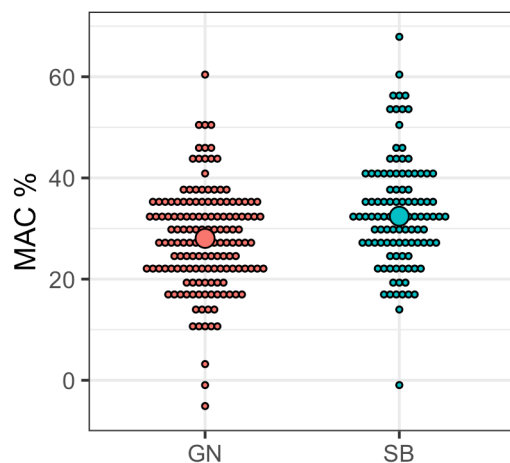

II\_453

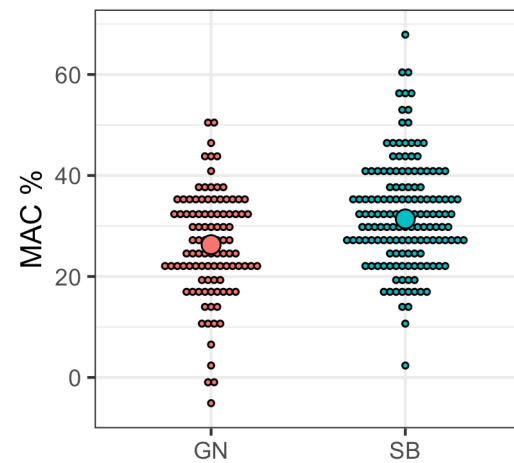

II\_657

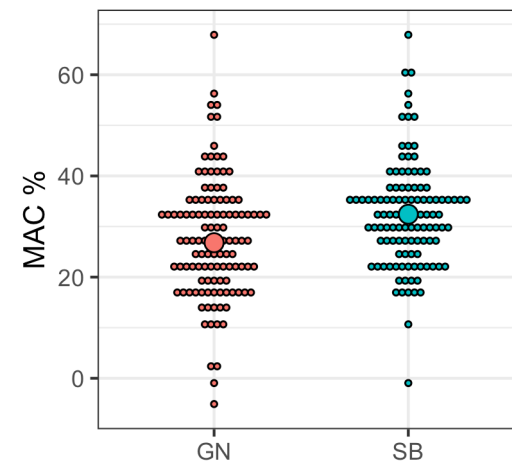

IV\_356

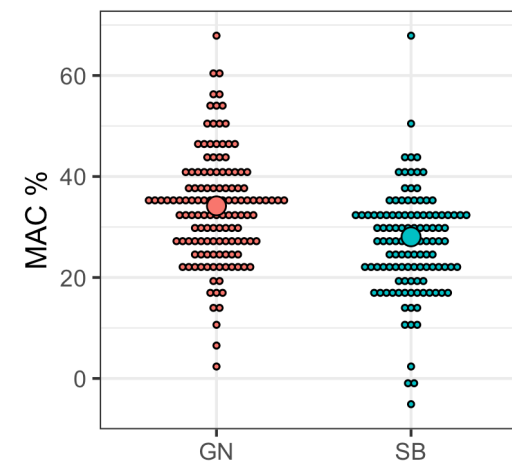

VII\_482

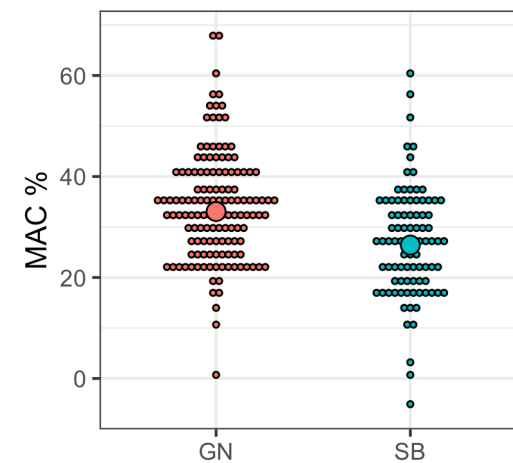

VII\_851

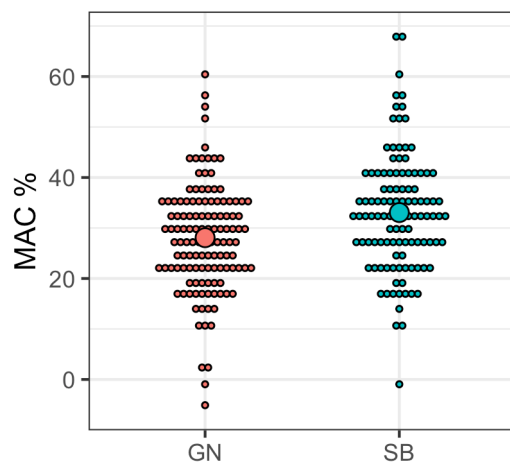

VIII\_489

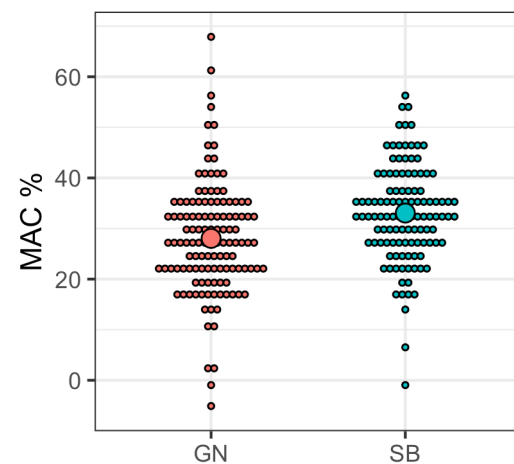

XI\_403

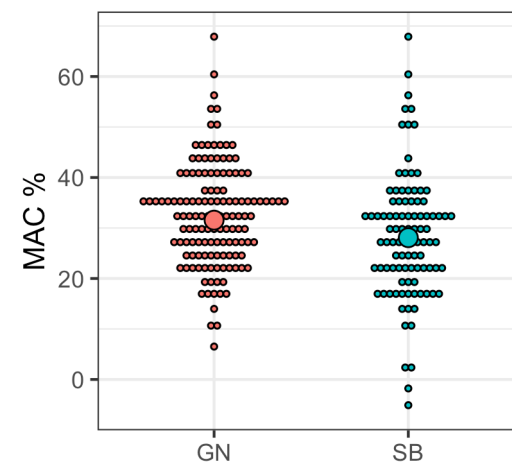

XII\_59

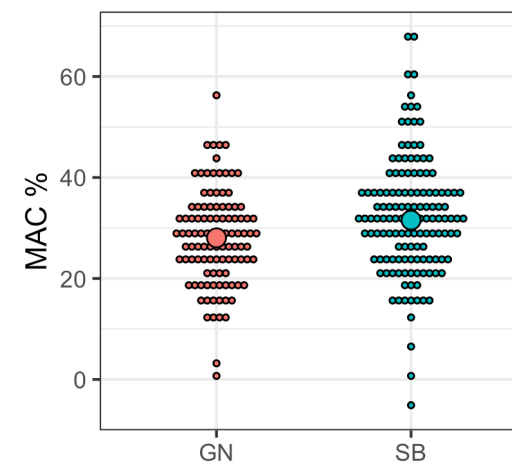

Supplement: Supplementary Figure 3 — QTL effect in population. Effect of each QTL according to parental inheritance. Each dot represents the phenotypic value of one individual and are colored according to their marker inheritance. Bigger points represent the mean of the population. [file Image_3.PDF]

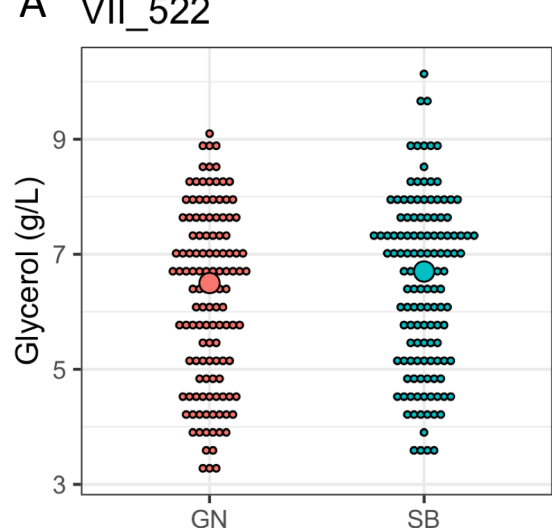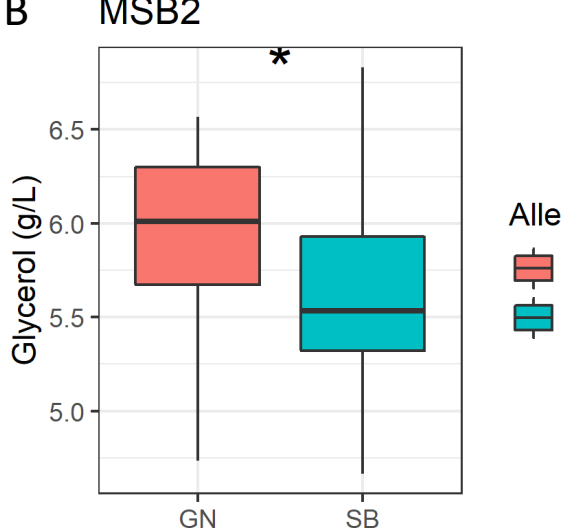

Supplement: Supplementary Figure 4 — Discrepancy for MSB2. (A) Effect of the marker associated to MSB2 in the offspring. Each dot represents the phenotypic value of one individual and are colored according to their marker inheritance. (B) Result of RHA test for MSB2. The represented value is from at least 5 biological replicates. The level of significance is indicated as follows: *p ≤ 0.1. **p ≤ 0.05. ***p ≤ 0.01. Solid lines of kinetic curves represent the mean and the shadow the standard error. [file Image_4.PDF]

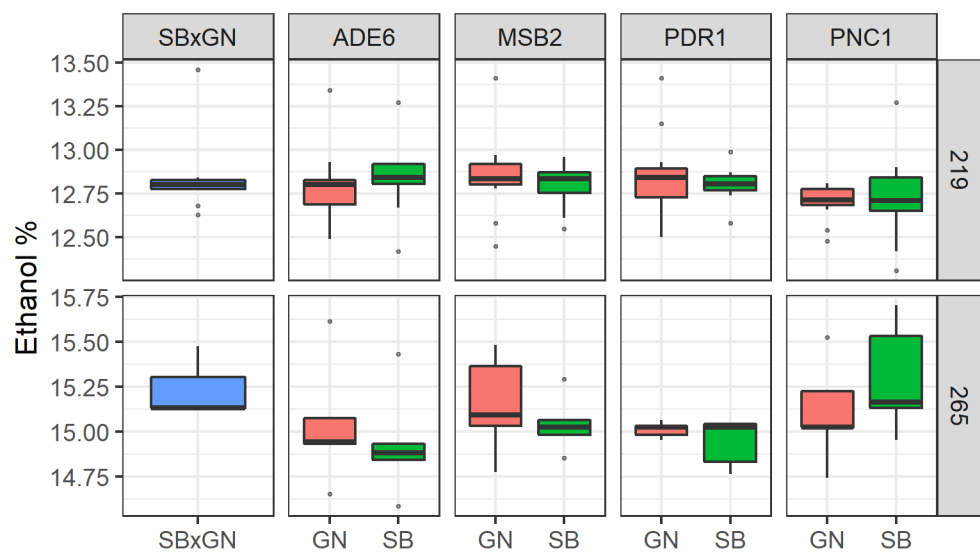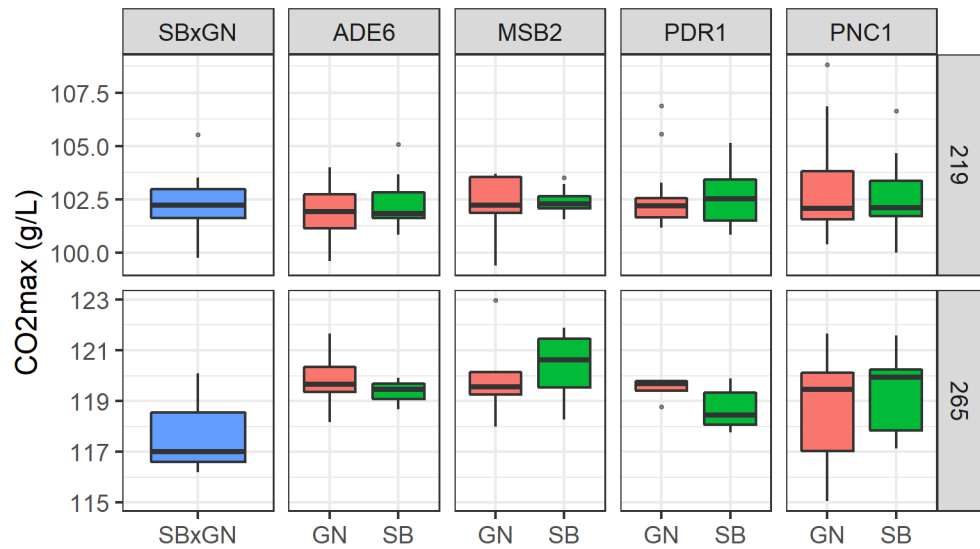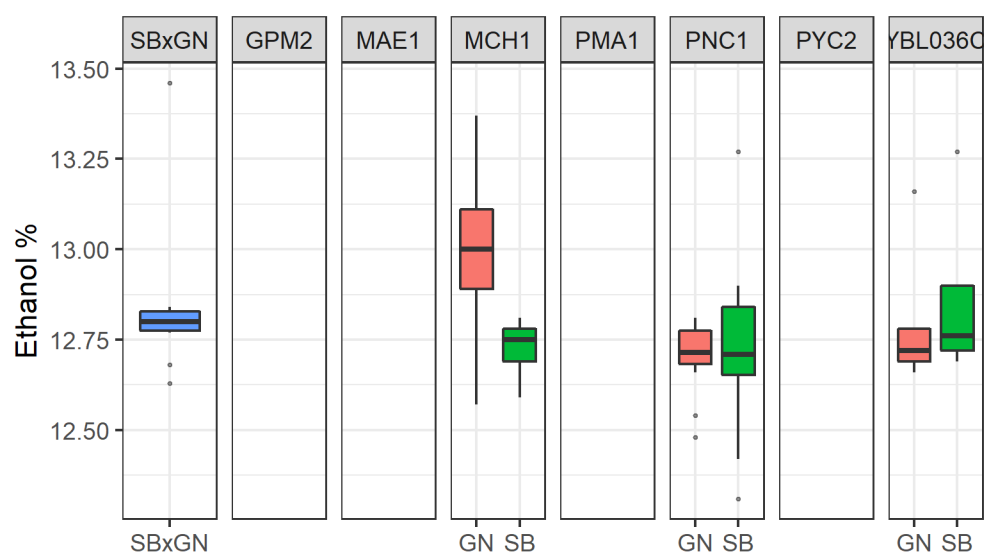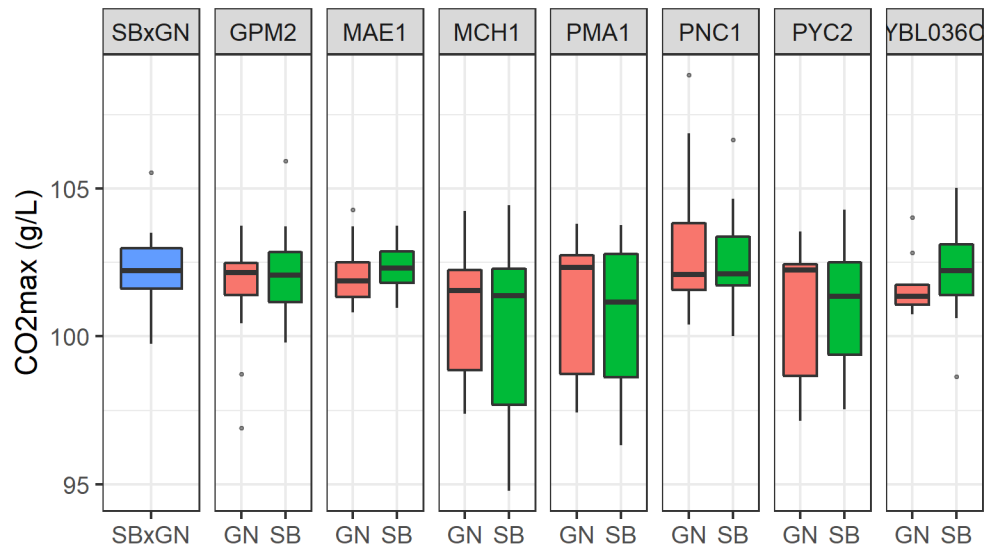

Supplement: Supplementary Figure 5 — Impact of targeted genes on CO2max and ethanol. Boxplot are colored according to the allele present in the hemizygous hybrids (blue = both, red = GN and green = SB) and represented the dispersion of at least five biological replicates. A Wilcoxon–Mann–Whitney test was applied to assess the significance of the phenotypic difference between hemizygotes. The level of significance is indicated as follows: *p ≤ 0.1, **p ≤ 0.05, ***p ≤ 0.01 and ****p ≤ 0.001. [file Image_5.PDF]

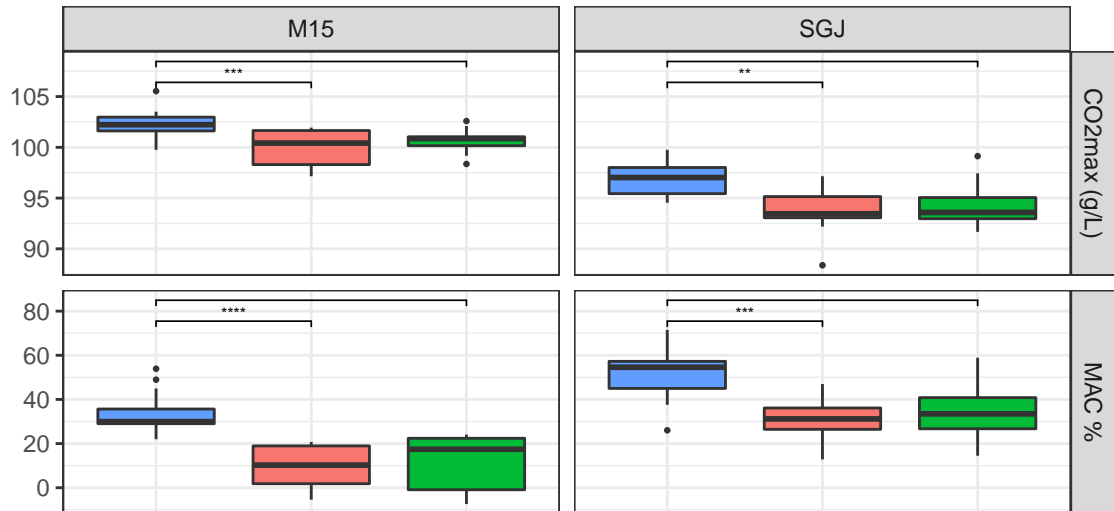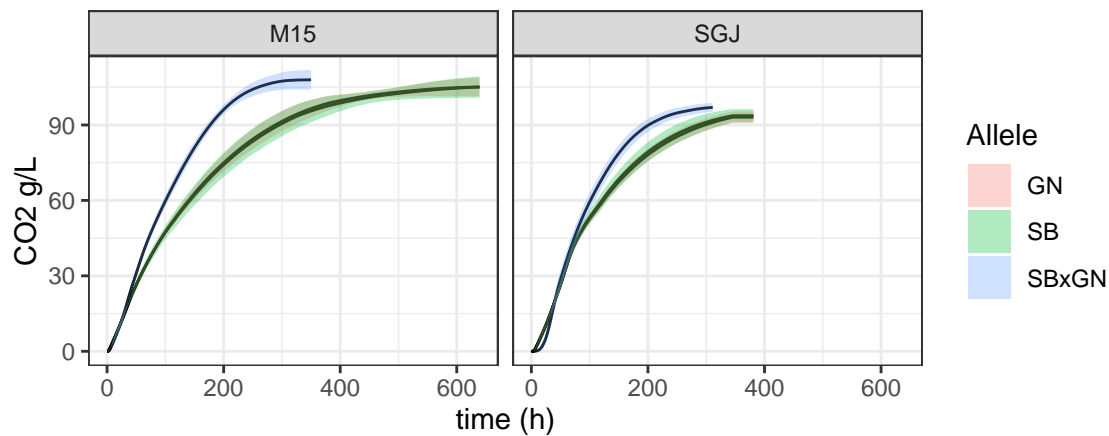

Supplement: Supplementary Figure 6 — SDH2 hemizygotes show a substantial haploinsufficiency according to media. The represented value is from at least 5 biological replicates. A Wilcoxon–Mann–Whitney test was applied to assess the significance of the phenotypic difference between wild type and hemizygote. The level of significance is indicated as follows: *p ≤ 0.1, **p ≤ 0.05, and ***p ≤ 0.01. Solid lines of kinetic curves represent the mean and the shadow the standard error. [file Image_6.PDF]

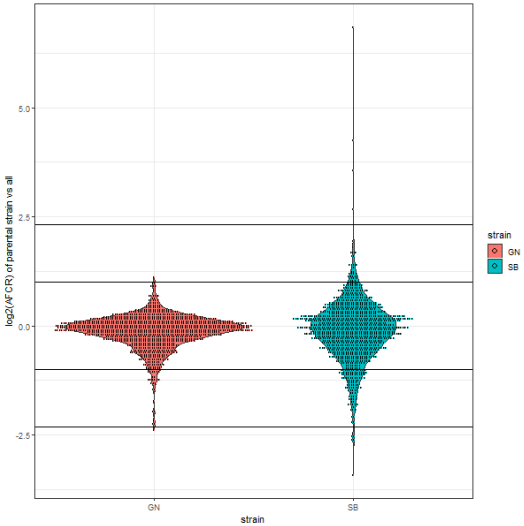

Supplement: Supplementary Figure 7 — SB proteome exhibit a strongest variability than GN respect to 24 others S. cerevisiae proteomes. The plot represents the distribution of the Abundance Fold Change Ratio (expressed in log2) of the strains SB and GN respect to the average values of 24 other strains. The variance of SB and GN computed for the 1,110 proteins indicated a highest variability of the SB proteome (F-test analysis <1·10−7). [file Image_7.PDF]

A

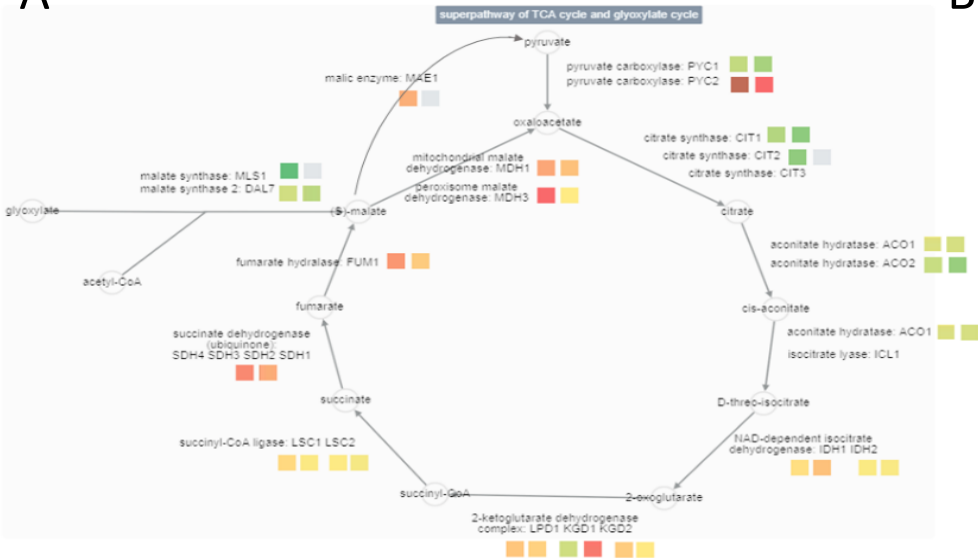

B

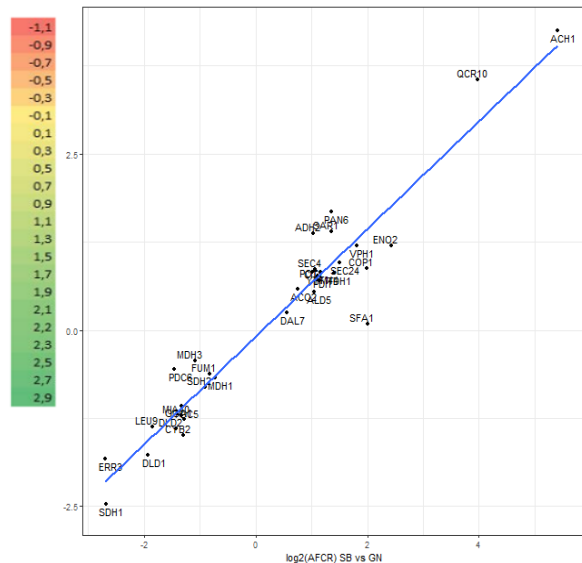

Supplement: Supplementary Figure 8 — Abundance of proteins belonging to the oxidative and reductive branches of TCA in SB respect to GN and others S. cerevisiae strains. (A) Abundance fold ratio of quantified proteins belonging to the TCA and the glyoxylate shunt; red and green colors indicated over and under expressed proteins in the SB strain vs. GN (left box) or vs. the average value of 24 S. cerevisiae strains (right box). (B) Correlation between the AFCR (log2) of SB vs. GN and SB vs. 24 S. cerevisiae strains for the commonly expressed proteins. [file Image_8.PDF]
