# Supplementary material for: Flor Yeasts Rewire the Central Carbon Metabolism During Wine Alcoholic Fermentation
Source: Front Fungal Biol. 2021 Oct 18;2:733513. doi: 10.3389/ffunb.2021.733513 (PMC10512321; doi:10.3389/ffunb.2021.733513)
Supplement: Supplementary File 2 — Hemizygotes construction. [file Presentation_2.PDF]

File S1 MOLECULAR TECHNIQUES

1. PCR TO AMPLIFY DELETION CASSETTES TO TRANSFORM

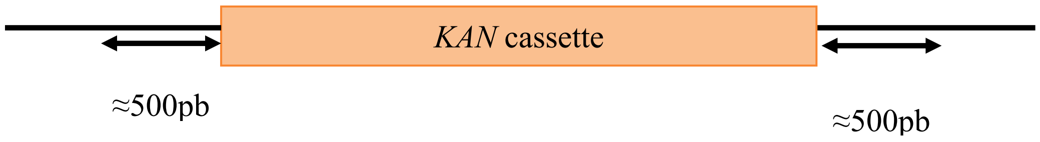

- Primers used:

Table 1. Primers used to amplify each gene specific deletion cassette.

Gene	Sequence	Type
ADE6	ATCGAGACTCGGTTGTGTCG	Forward
ADE6	GGTCCAGAAGCTAAGGCCTCC	Reverse
GPM2	CCAAGGCTCGACAAGGATGT	Forward
GPM2	CCGTGTGGTGCCCAATTTTC	Reverse
MAE1	TACTCTTCCCTAGGCGGTTT	Forward
MAE1	ATCCGGACATCACACCCAAC	Reverse
MCH1	TGGCAGAGTTTCAACAGCCA	Forward
MCH1	TGGGATACTTGGTGAATTCCGT	Reverse
PNC1	GTGGCACACAGGGTAATGAA	Forward
PNC1	TGCTCTTGAAATGAAAACGGAA	Reverse
PYC2	TGTCACTAACGACGTGTCCC	Forward
PYC2	CCCATTGGTTCTATTGGGCAG	Reverse
SDH2	ATTGCTGAGGTGCAAATGGC	Forward
SDH2	TGGTGTTCTCTTCTCATTGCT	Reverse
YBL036C	GAGCAACAGGTAACAGGGGA	Forward
YBL036C	GCGATGCTTTGGGAAAAGAGG	Reverse

2. PCR TO VERIFY TRANSFORMATION

Before transformation:

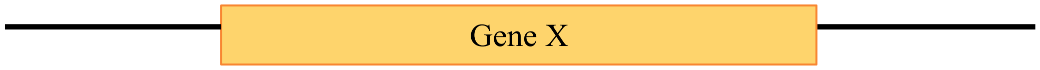

After transformation

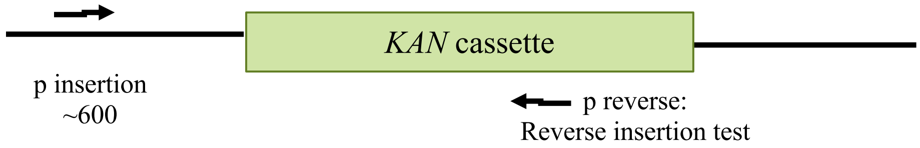

The PCR verification consist in using one primer at 600 pb (approx.) from the deleted gene loci and one primer that will anneal inside the *KAN* cassette. Thus, we verify the correct insertion of the KAN cassette in the right loci.

- Primers used:

Table 2. Primers used in the verification of the transformation.

Gene	Sequence	Type
All	CGGCGCAGGAACACTG	Anneals at the middle of the *KAN* cassette.
ADE6	CGACAAGAGTCCAAGAGGCAA	Insertion test
GPM2	CCACTCATTCAGAGGCTGTC	Insertion test
MAE1	TCGTGCATTGCAAGGTTTTT	Insertion test
MCH1	TAGTTCCACCGCCAATGGAT	Insertion test
PNC1	CCTCTTTCCCTACGATCCTCC	Insertion test
PYC2	TGTACTACAGGAAGCAGAAACA	Insertion test
SDH2	GGCTCAAGACCGTGAATGGA	Insertion test
YBL036C	GTTTGTTGGTGGGTTCTCGC	Insertion test

4. RFLP / qPCR

EITHER RFLP OR qPCR TO CHECK IF THE REMAINING ALLELE IN THE TRANSFORMANT IS SB OR GN.

Table 3. Primers used in the RFLP

Gene	Sequence	Type	Enzyme	Allele cut
MAE1	CGCTGCAGTGGACCAACT	RFLP forward	MboI	SB
MAE1	GCTAAGCACGATGTGTATGTTTTAT	RFLP reverse	MboI	SB
MCH1	TCTCAACCGTGGCAGAAACA	RFLP forward	PVUII	SB
MCH1	TGGGATACTTGGTGAATTCCGT	RFLP reverse	PVUII	SB
PNC1	TCTCCAGATACGATTATGATGTGCT	RFLP forward	CviKI-1	SB
PNC1	GGAGAGTGGTAGGTGTATGTTGA	RFLP reverse	CviKI-1	SB
PYC2	TGTCACTAACGACGTGTCCC	RFLP forward	MmeI	GN
PYC2	GATGAGCGTCTCTCCAGGTG	RFLP reverse	MmeI	GN

Table 4. Primers used in the qPCR

Gene	Sequence	Type
ADE6	TGACTTGGGTGCTAAATTCGATATTAGAAAGG	GN specific forward
ADE6	TGACTTGGGTGCTAAATTCGATATTAGAAAGA	SB specific forward
ADE6	TAACACCGACATCCGCAACA	qPCR reverse
GPM2	GACCCAACAGACCATAGAAACGAT	GN specific forward
GPM2	CCCAACAGACCATAGAAACGAC	SB specific forward
GPM2	ACAATCAGGCATGAAGATTCATCATATTGATT	qPCR reverse
SDH2	CCTTACTTACAGAGATCATCGTTTCCAA	GN specific forward
SDH2	CCTTACTTACAGAGATCATCGTTTCCAG	SB specific forward
SDH2	ACAGACCGGGTCATAGCATTG	qPCR reverse
YBL036C	GTTGTAATGCTATAAAAAAAAAGATCTTCGTT	GN specific reverse
YBL036C	GTTGTAATGCTATAAAAAAAAAGATCTTCGTA	SB specific reverse
YBL036C	AGTGGCACTTTATTGGCGGT	qPCR forward
